# ChatGPT’s Efficacy in Queries Regarding Polycystic Ovary Syndrome and Treatment Strategies for Women Experiencing Infertility

**DOI:** 10.3390/diagnostics14111082

**Published:** 2024-05-22

**Authors:** Belgin Devranoglu, Tugba Gurbuz, Oya Gokmen

**Affiliations:** 1 Department of Obstetrics and Gynecology, Zeynep Kamil Maternity/Children, Education and Training Hospital, Istanbul 34480, Turkey; 2 Department of Gynecology and Obstetrics Clinic, Medistate Hospital, Istanbul 34820, Turkey; drtugbagurbuz@gmail.com; 3 Department of Gynecology, Obstetrics and In Vitro Fertilization Clinic, Medistate Hospital, Istanbul 34820, Turkey; gokmenoya@hotmail.com

**Keywords:** PCOS, artificial intelligence, ChatGPT_4_, medical queries

## Abstract

This study assesses the efficacy of ChatGPT-4, an advanced artificial intelligence (AI) language model, in delivering precise and comprehensive answers to inquiries regarding managing polycystic ovary syndrome (PCOS)-related infertility. The research team, comprising experienced gynecologists, formulated 460 structured queries encompassing a wide range of common and intricate PCOS scenarios. The queries were: true/false (170), open-ended (165), and multiple-choice (125) and further classified as ‘easy’, ‘moderate’, and ‘hard’. For true/false questions, ChatGPT-4 achieved a flawless accuracy rate of 100% initially and upon reassessment after 30 days. In the open-ended category, there was a noteworthy enhancement in accuracy, with scores increasing from 5.53 ± 0.89 initially to 5.88 ± 0.43 at the 30-day mark (*p* < 0.001). Completeness scores for open-ended queries also experienced a significant improvement, rising from 2.35 ± 0.58 to 2.92 ± 0.29 (*p* < 0.001). In the multiple-choice category, although the accuracy score exhibited a minor decline from 5.96 ± 0.44 to 5.92 ± 0.63 after 30 days (*p* > 0.05). Completeness scores for multiple-choice questions remained consistent, with initial and 30-day means of 2.98 ± 0.18 and 2.97 ± 0.25, respectively (*p* > 0.05). ChatGPT-4 demonstrated exceptional performance in true/false queries and significantly improved handling of open-ended questions during the 30 days. These findings emphasize the potential of AI, particularly ChatGPT-4, in enhancing decision-making support for healthcare professionals managing PCOS-related infertility.

## 1. Introduction

Polycystic ovary syndrome (PCOS) is a multifaceted endocrine pathology affecting no less than 10% of women in their reproductive years, underscoring its status as one of the predominant factors contributing to female infertility [1]. It is characterized by a spectrum of some symptoms, including but not limited to oligomenorrhea, hyperandrogenism, and polycystic ovaries [2,3,4]. Given its multifaceted nature, the management of infertility in women with PCOS requires a comprehensive approach, of which ovulation induction is a cornerstone [5,6,7]. Advancements in assisted reproductive technology and pharmacological interventions have significantly improved these patients’ prognoses, yet the quest for optimal treatment strategies remains ongoing [8].

The development of artificial intelligence (AI) technology has witnessed substantial advancements across numerous sectors, with a particularly notable exemplar being the sophisticated language models such as ChatGPT_4_ (by OpenAI) [9,10]. This language model, distinguished for its ability to generate text of high quality in response to diverse prompts, utilizes training data to construct neural networks. These networks are adept at discerning patterns and associations amongst words and phrases, enabling the model to respond intelligently to various inquiries and commands [11]. It offers significant potential in aggregating and analyzing vast research data, thereby identifying effective treatment paradigms and novel therapeutic targets. Specifically, in the context of ovulation induction, it can play a pivotal role in investigating treatment protocols for individual patient profiles, thus maximizing the chances of successful pregnancy while minimizing the risks of treatment-related complications [12,13].

The collaboration of industry and academia has confirmed using data in informing data-driven decisions for assisted reproductive technology [14]. The effectiveness of the models can be enhanced by accessing larger, multi-center datasets. These should encompass diverse patients and reflect the unique aspects of practices globally [15]. Grünebaum et al. highlight ChatGPT’s potential to provide preliminary information across a broad spectrum of clinical queries, though they caution about its limitations, including data currency and the lack of source citation capabilities, which might mislead users [16]. Similarly, Allahqoli et al. demonstrated ChatGPT’s robust diagnostic and management capabilities in obstetrics and gynecology, albeit with an acknowledgment of inherent biases and the need for careful consideration of ethical issues surrounding its use [17]. The technology’s potential extends to competitive scenarios, as evidenced by a study by Li et al., wherein ChatGPT outperformed human candidates in a virtual objective structured clinical examination [18]. This suggests not only a proficiency in providing accurate and contextually appropriate responses but also an ability to do so more efficiently than human counterparts. While these findings are promising, Lee and Kim note that the ongoing challenge remains the verification of information and the ethical implications associated with AI in medical settings [19]. The potential for ChatGPT and similar technologies in obstetrics and gynecology is significant, yet the integration of such AI tools into clinical practice requires careful navigation of both technical and ethical landscapes to ensure they augment rather than undermine professional healthcare delivery.

The integration of ChatGPT_4_ into the management of PCOS-related infertility represents a promising frontier in reproductive medicine. Our research aims to assess the accuracy and reliability of ChatGPT_4_ in providing information on PCOS treatment options, with the objectives of verifying the model’s responses against current medical guidelines and observing its performance consistency over time.

## 2. Materials and Methods

### 2.1. Study Design

The study utilized a cross-sectional design to evaluate the capabilities of ChatGPT_4_, a sophisticated AI language model, in understanding and providing treatment protocols for PCOS-related infertility. The model was queried using a variety of question formats—true/false, multiple-choice, and open-ended—following the latest 2023 guidelines. The study aimed to assess ChatGPT_4_’s effectiveness as a support tool in decision-making within clinical environments, concentrating on its precision, dependability, and capability to serve as a learning resource for medical professionals. The research did not engage with human/animal participants, thus eliminating the need for ethical consent and approval. All methods adhered to the ethical standards and principles outlined in the Declaration of Helsinki.

### 2.2. Study Design and Query Types

We did not prompt any specific order to ChatGPT_4_ before or after asking the queries and answering sequence. The queries, designed by the authors and based on current guidelines, were divided into three categories: true/false (170 queries): The assessments were intended to evaluate AI’s capacity for correctly confirming or debunking claims. Open-ended (165 queries): Each open-ended query was accompanied by a specific rubric detailing the key points that should be included in a correct response, aiming to ensure consistency among evaluators in scoring the responses based on completeness and accuracy. The correct answers were not predetermined so we utilized expert review to evaluate the responses generated by ChatGPT_4_. This method evaluated AI’s acknowledgment in understanding and generating responses that align with the intent and content of the questions asked. Multiple-choice (125 queries): The queries were designed to measure AI’s skill in selecting the appropriate response from a range of options, based on established guidelines. They were analyzed by assessing whether the selected answer matched the predetermined correct answer. Focusing on PCOS, the complexity of each question was considered, and ChatGPT’s responses were documented initially and then again after 30 days to assess consistency.

The queries were chosen through the guidelines and designed collaboratively by the authors to encompass a broad range of standard and complex scenarios encountered in PCOS management. The difficulty of the questions was chosen from the guidelines and determined by consensus among the authors, based on their clinical experience and the expected knowledge level of a general medical practitioner in gynecology. The scope spanned diagnostic standards, therapeutic approaches, monitoring procedures for PCOS, and advice for patients, offering a thorough evaluation of AI’s performance. In this research, the analysis was carried out by two evaluators, resulting in a Cohen’s kappa coefficient of 0.87 for binary questions, indicating excellent agreement (*p* < 0.001).

### 2.3. Analysis of ChatGPT_4_ Responses

This research, conducted from 1 January to 30 January 2024, analyzed AI’s answers using the Likert scale method, a popular technique for measuring attitudes and opinions. While the Likert scale is a widely accepted tool, its use in assessing the correctness of AI-generated text does present limitations, particularly in capturing the nuanced accuracy of responses. Two experts in obstetrics and gynecology evaluated each answer’s precision, relevance, and guideline compliance. Prior to the evaluation phase, all evaluators underwent a standardized training session to align their understanding of the assessment criteria, ensuring consistency in rating the responses. This involved a six-point scale for assessing response accuracy (ranging from 1 for utterly incorrect to 6 for entirely correct). The analysis was structured around two main aspects: accuracy, which involved calculating the proportion of correct answers, and completeness, which examined AI’s capacity to provide comprehensive responses across similar queries consistently and the scope of AI’s replies in terms of detail and coverage. To mitigate potential biases and enhance the robustness of our findings, a blind evaluation process was implemented. Evaluators were unaware of the specifics of the queries that generated the responses they were assessing. Inter-rater reliability was periodically checked to ensure consistency among evaluators, with discrepancies discussed and resolved through consensus. Prior to its application, a pilot test was conducted to verify its effectiveness in our specific context, particularly in measuring the correctness and relevance of AI-generated answers. These steps, from evaluator training to the adaptation and testing of the Likert scale, underline our commitment to providing a rigorous and unbiased evaluation of AI-generated text. These measures not only demonstrate the reliability of our evaluation method but also reflect our dedication to continuously improving our research methodologies. Inter-rater reliability was initially assessed only for true/false questions due to their binary and unambiguous nature, which provided a clear basis for preliminary reliability analysis.

### 2.4. Data Analysis

All statistical procedures were carried out using the SPSS version 24 software provided by IBM Co., Chicago, IL, USA. The data gathered were synthesized by employing an array of descriptive statistics. The median, defining the central value in a hierarchically organized dataset alongside the mean, is central among these and indicative of the arithmetic average. A chi-squared analysis was executed to probe the stability of ChatGPT_4_’s responses to inquiries about PCOS across temporal intervals. Responses were rated on a six-point Likert scale for accuracy and completeness. Statistical tests included chi-squared for stability over time, the Mann–Whitney U test for comparisons between two groups, and Kruskal–Wallis and Wilcoxon signed-rank tests for multiple groups and paired samples, respectively. Bidirectional tests were utilized throughout, with a *p*-value threshold set below 0.05. This methodological approach is instrumental in ascertaining whether a notable discrepancy exists in the ratio of accurate-to-inaccurate responses, contrasting initial replies with those produced after a 30-day interlude.

## 3. Results

As seen in Table 1, the effectiveness and consistency of ChatGPT_4_’s responses to questions regarding PCOS were evaluated over one month using open-ended and multiple-choice question formats. The analysis focused on two primary metrics: the accuracy and completeness of the responses, which were assessed through Likert scoring.

### 3.1. True/False Queries

Furthermore, as part of the study, ChatGPT_4_ was asked 170 true/false format questions related to PCOS. ChatGPT_4_ responded to all questions with 100% accuracy in the initial assessment. This achievement was maintained in a repeat evaluation 30 days later, where ChatGPT accurately answered all questions again, demonstrating consistent performance over time for this question type.

### 3.2. Open-Ended Queries

A noticeable improvement in accuracy scores from the initial to the 30th day was observed for open-ended questions. The average accuracy score at the outset was 5.53 ± 0.887, which increased to 5.88 ± 0.43 by the end of the month. This result signifies a substantial improvement in response accuracy over time. Regarding completeness, the initial responses had an average completeness score of 2.35 ± 0.581, which rose to 2.92 ± 0.29 by the 30th day, indicating a significant enhancement in the thoroughness of the responses within a month.

### 3.3. Multiple-Choice Queries

For multiple-choice questions, the accuracy scores remained relatively stable, starting at 5.96 ± 0.44 and slightly decreasing to 5.92 ± 0.63 by the end of the month, demonstrating no significant change over time. The completeness scores for multiple-choice responses showed minimal variation; the initial scores were 2.98 ± 0.18, and by the 30th day, the scores were 2.97 ± 0.25, indicating a consistent quality in the completeness of the responses.

### 3.4. Performance for Difficulty Levels

**Figure 1:**
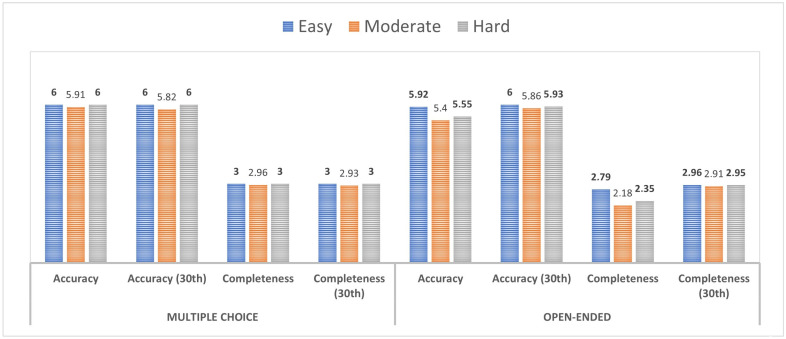
Comparison of ChatGPT_4_’s responses to multi-choice and open-ended queries in terms of difficulty levels. The bold numbers in the figure represent the highest performance scores for different criteria in each category (Easy, Moderate, Hard).

For multiple-choice questions, accuracy was consistently high across all difficulty levels, with 6.00 for both easy and hard and a marginally lower score of 5.91 for moderate difficulty. This pattern persisted on the 30th day, with scores remaining at 6.00 for easy and hard levels and a slight decrease to 5.82 for moderate difficulty (Figure 1). Completeness was stable across difficulty levels at 3.00, with a negligible decrease observed in moderate difficulty (2.96) and at the 30th day for moderate difficulty (2.93). Open-ended tasks showed a variable pattern. Accuracy scores decreased with increasing difficulty: 5.92 (easy), 5.40 (moderate), and 5.55 (hard). However, on the 30th day, the scores were 6.00 (easy), 5.86 (moderate), and surprisingly higher at 5.93 (hard). Completeness scores for open-ended tasks were lower than those for multiple-choice tasks, with the highest score being 2.79 for easy difficulty, dropping to 2.18 for moderate and increasing slightly to 2.35 for hard. On the 30th day, the scores saw an upward trend, resulting in 2.96 (easy), 2.91 (moderate), and 2.95 (hard).

## 4. Discussion

The current analysis is precious as it appears to be the first to investigate the power and effectiveness of AI-ChatGPT_4_ in providing accurate and comprehensive responses to questions related to PCOS and treatment strategies for women experiencing infertility. This condition requires a nuanced understanding and management. Our findings offer insightful implications for applying AI-ChatGPT_4_ in providing accurate responses to queries about PCOS because its performance exhibited significant traits regarding compliance with guidelines for different difficulty levels. The improvements and consistencies observed across different queries—open-ended, multiple-choice, and true/false—highlight ChatGPT_4_’s evolving capabilities and potential role in health education and information dissemination.

Recent scholarly explorations underscore the burgeoning utility of AI, notably ChatGPT, in the obstetrics and gynecology domain, albeit clinical validation remains nascent. Notably, comparative analyses have positioned ChatGPT’s proficiency above that of seasoned human counterparts in examinations, as highlighted in pertinent studies [17,20]. A seminal publication delineated the efficacious deployment of ChatGPT in responding to a wide array of inquiries posited by quartets of clinicians, with the discourse encapsulated in quotations subjected to expert critique [16]. Suhag et al. delineated ChatGPT’s potential in augmenting differential diagnosis formulation and guiding patients and medical cohorts in navigating the complexities of rare prenatal conditions [20]. The discourse extends to the exploration by Santo et al. [21], elucidating it as an instrumental resource in disseminating intelligible information to laypersons during unanticipated labor scenarios. Allahqoli et al. embarked on a comparative inquiry into its diagnostic and management acumen across a spectrum of cases compared to a gynecologist’s expertise [17]. Its accuracy was evidenced at 90% across 30 diverse cases, underscoring its competency in delivering articulate, informed responses, with an inherent capacity to rectify initial diagnostic inaccuracies upon subsequent elucidations.

All recent studies have indicated that AI exhibits varying levels of accuracy and success across different medical specialties, with performance rates fluctuating [22,23,24]. However, the outcomes of our study on PCOS surpass the average results documented in the literature, indicating a much higher level of precision and efficacy. The consistent 100% accuracy in responding to true/false format questions related to PCOS, both initially and after 30 days, underscores ChatGPT_4_’s capability to accurately discern factual correctness in statements. This consistency is particularly noteworthy as it suggests a robust understanding of PCOS-related facts, which is critical for misinformation prevention and accurate public health communication. Despite these strengths, several limitations of our study must be addressed to fully appreciate the scope and reliability of our findings. The inherent biases in AI’s training data could skew results and limit the generalizability across different populations or subtypes within PCOS. Additionally, the subjective nature of evaluating open-ended responses introduces a level of variability that could affect the reliability of our conclusions. Importantly, our study did not incorporate dynamic learning from the queries during the study period, which could have provided deeper insights into AI’s adaptability and long-term learning capabilities. Another medical study by Campbell et al. reported that ChatGPT_4_ can produce suitable responses to inquiries [25]. However, this study also highlighted a notable occurrence of AI-generated inaccuracies, colloquially referred to as “hallucinations”, casting doubts on AI’s dependability as an educational instrument. This finding is complemented by the research conducted by Deniz et al., who observed a 6% enhancement in the correction of errors juxtaposed with a 12% decrease in the accuracy of first answers, underlining the fluctuating yet inconsistent efficacy [24]. These issues underscore the need for ongoing research to mitigate AI’s limitations, such as improving data diversity and developing mechanisms for dynamic learning, to enhance the reliability of AI-driven educational content in different medical fields.

Furthermore, Deniz et al. reported negligible variations in accuracy by time, aligning with the mild concurrence and reliability documented in preceding research [25,26], thus emphasizing AI’s variable performance metrics. In our analysis, the substantial improvement in accuracy scores from the initial assessment to the 30th day for open-ended queries indicates that ChatGPT_4_’s responses became more precise over time. This could be attributed to its learning algorithms, which adjust based on interaction and feedback, enhancing the model’s performance. The significant increase in completeness scores also suggests that ChatGPT_4_ can provide more detailed and comprehensive answers as it ‘learns’ from its interactions. The stability of accuracy and completeness scores in multiple-choice queries suggests that ChatGPT_4_ already possesses a high level of understanding and capability in handling this type of question from the outset. The slight decrease in accuracy scores might not indicate a degradation in performance but rather the variability inherent in any learning system when dealing with nuanced content over time. While multiple-choice task performance remained relatively unaffected by difficulty, open-ended queries showed more sensitivity to difficulty, with a notable impact on accuracy and completeness. An improvement pattern in open-ended tasks was observed at the 30th iteration, particularly for the hard difficulty level, which may imply a learning effect or increased familiarity with the task requirements.

While this study demonstrates the potential for AI in medical query resolution, allows for observing ChatGPT_4_’s performance over time, and provides insights into its learning capabilities and adaptability within PCOS-related queries, it is not without limitations. These include potential biases in AI’s training data, the subjective nature of evaluating open-ended responses, and the absence of dynamic learning from the queries during the study period. These factors could influence the generalizability of the results and the consistency of AI’s performance over different datasets. While the improvement in open-ended query responses suggests learning over time, the mechanism behind this was not explored, leaving questions about the factors contributing to the enhanced performance. This oversight highlights the need for future research to mitigate such inaccuracies and improve the reliability of AI-driven educational content. The cutoff date for the knowledge base used to train ChatGPT_4_ imposes certain constraints on the generalizability and long-term relevance of our findings. As the body of knowledge continues to evolve, the responses generated by the model may not reflect the most current information or emerging trends. This limitation underscores the importance of periodic updates to AI’s training data to maintain their accuracy and relevance.

## 5. Conclusions

In summary, our study demonstrates the potential of AI through ChatGPT_4_ in providing precise and comprehensive responses to PCOS-related questions, consistently achieving high accuracy across various question formats and difficulty levels. Notably, AI’s performance in true/false queries maintained a remarkable 100% accuracy rate both initially and after a 30-day interval, underscoring its reliability. Despite its strengths, limitations such as potential biases in training data, the subjective nature of open-ended response evaluations, and the absence of dynamic learning from interactions during the study highlight areas for future improvement. These insights are vital for integrating AI tools like ChatGPT_4_ into medical education and patient interaction settings, though they must be deployed judiciously alongside human expertise.

## Figures and Tables

**Table 1:** ChatGPT’s responses to open-ended and multiple-choice queries.

Query Types	Score Metrics		Mean ± SD (*Range*)	*p*-Value
Open-Ended Queries (*n*: *165*)	Accuracy	*Initial*	5.53 ± 0.88 (*3*–*6*)	0.0001
		*30th day*	5.88 ± 0.43 (*5*–*6*)	
	Completeness	*Initial*	2.35 ± 0.58 (*2*–*3*)	0.0001
		*30th day*	2.92 ± 0.29 (*2*–*3*)	
Multiple Choice Queries (*n*: *125*)	Accuracy	*Initial*	5.96 ± 0.44 (*5*–*6*)	0.566
		*30th day*	5.92 ± 0.63 (*5*–*6*)	
	Completeness	*Initial*	2.98 ± 0.18 (*2*–*3*)	0.705
		*30th day*	2.97 ± 0.25 (*2*–*3*)	

The table summarizes the evaluation of ChatGPT‘s performance on different queries over an initial period and after 30 days. Scores are presented as mean ± standard deviation (SD) with ranges in parentheses. The *p*-value indicates the statistical significance of the difference between the initial and 30th-day scores.

## Data Availability

The data supporting this study’s findings are available from the corresponding author upon reasonable request.

## List of Acronyms

AI	Artificial Intelligence
PCOS	Polycystic Ovary Syndrome
ChatGPT	Chat Generative Pre-Trained Transformer
SPSS	Statistical Package for the Social Sciences
IBM	International Business Machines Corporation
USA	United States of America
IACUC	Institutional Animal Care and Use Committee
RCT	Randomized Controlled Trial
